# Time Course of Renal Transcriptomics after Subchronic Exposure to Ochratoxin A in Fisher Rats

**DOI:** 10.3390/toxins13030177

**Published:** 2021-02-26

**Authors:** Laura Pastor, Ariane Vettorazzi, Elizabeth Guruceaga, Adela López de Cerain

**Affiliations:** 1 Department of Pharmacology and Toxicology, Faculty of Pharmacy and Nutrition, University of Navarra, CIFA Building, c/Irunlarrea 1, E-31008 Pamplona, Spain; lpastorcastro@gmail.com (L.P.); acerain@unav.es (A.L.d.C.); 2 IdiSNA, Navarra Institute for Health Research, E-31008 Pamplona, Spain; eguruce@unav.es; 3 Bioinformatics Platform, Center for Applied Medical Research (CIMA), University of Navarra, E-31008 Pamplona, Spain

**Keywords:** ochratoxin A (OTA), sex differences, gene expression, metabolism, transporters, Fisher rats

## Abstract

The mycotoxin ochratoxin A (OTA) is a potent nephrocarcinogen, mainly in male rats. The aim of this study was to determine the time course of gene expression (GeneChip^®^ Rat Gene 2.0 ST Array, Affymetrix) in kidney samples from male and female F344 rats, treated daily (p.o) with 0.50 mg/kg b.w. (body weight) of OTA for 7 or 21 days, and evaluate if there were differences between both sexes. After OTA treatment, there was an evolution of gene expression in the kidney over time, with more differentially expressed genes (DEG) at 21 days. The gene expression time course was different between sexes with respect to the number of DEG and the direction of expression (up or down): the female response was progressive and consistent over time, whereas males had a different early response with more DEG, most of them up-regulated. The statistically most significant DEG corresponded to metabolism enzymes (*Akr1b7*, *Akr1c2*, *Adh6* down-regulated in females; *Cyp2c11*, *Dhrs7*, *Cyp2d1*, *Cyp2d5* down-regulated in males) or transporters (*Slc17a9* down-regulated in females; *Slco1a1* (OATP-1) and *Slc51b* and *Slc22a22* (OAT) down-regulated in males). Some of these genes had also a basal sex difference and were over-expressed in males or females with respect to the other sex.

## 1. Introduction

Ochratoxin A (OTA) is a mycotoxin produced by fungi of the genera *Aspergillus* and *Penicillium,* which contaminate vegetal products and enter the food chain [1]. The OTA-target organ is the kidney, but it is also considered hepatotoxic, immunosuppressive, neurotoxic, and teratogenic [1,2]. The main concern is its nephrocarcinogenicity, as it is considered one of the most potent renal carcinogens in rodents [3]. Few carcinogenic studies have been performed in rodents, and it has been demonstrated that male animals are more sensitive than females with respect to the tumoral effect of OTA (Table 1) [4,5,6,7]. In humans, it is classified as a probable human carcinogen [8] and reasonably anticipated to be a human carcinogen [9]. 

In spite of the great number of studies that have been performed to date, the mechanisms of action of its nephrotoxicity and/or carcinogenicity are not completely understood (for a review, see [10,11,12]). Six hypotheses were considered for its carcinogenic mode of action (MoA) by WHO in 2008 [13] and can still be considered as valid: (a) Genotoxicity from direct interaction of OTA or a reactive metabolite with DNA, (b) Generation of tumors secondary to chronic renal toxicity and compensatory cell proliferation, (c) Generation of tumors secondary to inhibition of phenylalanine-tRNA synthetase and protein synthesis, (d) Disruption of cell-cell signaling pathways and the process of cell division, (e) Alteration of intracellular calcium homeostasis, and (f) Mitochondrial dysfunction leading to oxidative stress and indirect induction of DNA damage. Some authors suggested that OTA is a genotoxic carcinogen by induction of oxidative damage to DNA coupled with DNA adducts via quinone formation [14,15], while others pointed to a complex network of interacting mechanisms (protein synthesis inhibition, oxidative stress, modulation of transcription factors, such as the nuclear factor E2-related factor 2 (Nrf2), the hepatocyte nuclear factor-4α (HNF4α), or the nuclear factor NF-kappaB (NF-κB), and activation of mitogen-activated protein kinases (MAPKs) and calcium homeostasis pathways) [16,17,18]. Finally, inhibition of histone acetyltransferases, disruption of mitosis, cell proliferation, and genetic instability have also been proposed as events in OTA MoA [19]. Overall, it seems that OTA carcinogenicity might be due to a combination of several mechanisms, including direct genotoxicity, oxidative stress, or epigenetic factors [10]. Regarding the risk assessment, a direct genotoxic mechanism (DNA binding of OTA) is considered as a non-threshold mechanism, while indirect damage to DNA or non-genotoxic mechanisms is considered to have a threshold, and thus safe health-based guidance values can be established. In case of no clear information, a non-threshold mechanism of action has been assumed by some regulatory agencies [20].

Whole-genome gene expression analysis has been considered a powerful tool for understanding or unraveling the mechanism of action of certain compounds [21]. Indeed, several transcriptomic studies have been carried out with OTA either in vitro or in vivo with different purposes (for a review, see [22]). The gene expression time-course of repeated OTA exposure has been specifically addressed in a few studies, and all of them have been carried out only in males. Gene expression in kidney and liver of daily treated male F344 rats was analyzed at five intervals (7 days, 21 days, 4 months, 7 months, and 12 months), and it was found that gene expression profiles were the most similar at day 7 and month 12 and that the responses were specific in kidney versus liver [23]. Moreover, a time-dependent increase of OTA concentration in kidney and liver of male F344 rats treated with OTA for 7 or 21 days was obtained but without differences between both tissues; on the contrary, the number of differentially expressed genes (DEG) was much higher in kidney than in liver at both time points [24]. In both studies, a general tendency towards gene expression down-regulation was observed, as in other toxicogenomic studies with OTA [25]. In addition, a clear specific response of the kidney to OTA injury could be distinguished in male animals. 

The aim of the present study was to study the time-course of gene expression profile in the kidney of male and female OTA-treated animals to see if there was any specific key step at the early stages of intoxication. For that purpose, gene expression was studied in kidney samples obtained from male and female F344 rats administered with a daily oral dose of 0.50 mg/kg b.w. (body weight) of OTA for 7 or 21 days. This dose was selected because it is slightly higher than the carcinogenic dose in rats (see Table 1), and it is a dose that has shown toxic effects in short-term studies performed by us and other authors [24,25]. 

## 2. Results

### 2.1. Gene Expression in Control Rats

**Figure 1:**
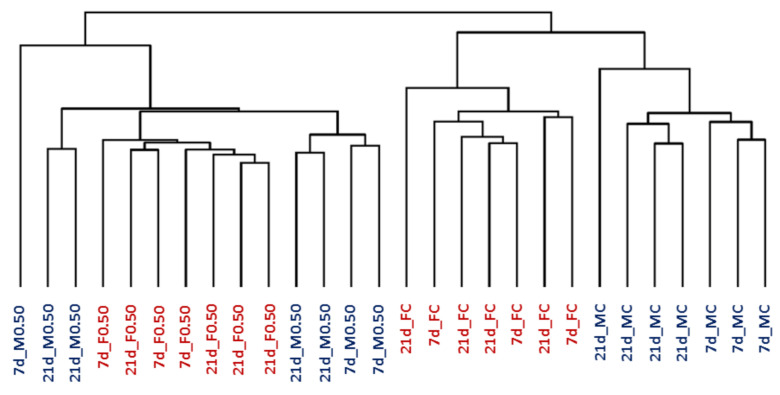
Hierarchical clustering analysis of kidney samples from male and female rats (M, in blue; F, in red). They were treated for 7 or 21 days (7d; 21d) with 0.50 mg OTA/kg b.w. (0.50) or with the vehicle (NaHCO_3_) (C). OTA, ochratoxin A; b.w., body weight.

The hierarchical clustering analysis (HCA), which was performed for each time point with all the genes present on the GeneChip, showed that animals were grouped in two main clusters: control groups and OTA-treated animals. In addition, a clear different expression was observed between females and males in the control groups (Figure 1).

DEG between male and female control animals was analyzed at each time point in order to determine basal sex-dependent expression differences. The age of the animals of this study sacrificed after 7 and 21 days were 13- and 15-week-old, respectively. The total number of DEGs was 876 in 7 days group and 950 in 21 days group, but of all of them, only 69 genes at 7 days and 82 genes at 21 days had logFC (logarithm Fold Change) higher than 1.5 or less than −1.5. Interestingly, 51 of these genes were common at both time points, so they could be considered as well-established sex-dependent genes and are presented in Table 2. Positive values of logFC mean that the gene is more expressed in males than in females, and negative values of logFC indicate that the gene is more expressed in females than in males. As shown in Table 2, many of the genes encoded for phase I and phase II enzymes or transporters: males had higher expression levels of *Dhrs7* (dehydrogenase/reductase), *Cyp2c11, Cyp2d1*, *Cyp2d5*, *Cyp4a2* (cytochromes), *Sult1b1* (sulfotransferase), *Akr1c12|1*, *Akr1c12* (aldo-keto reductases), *Slco1a1*, *Slc22a22,* and *Slc51b* (transporters); females showed higher expression of *Adh6*, *Adh1* (alcohol dehydrogenases), *Akr1b7*, *Akr1c2* (aldo-keto reductases), *Slc17a9*, *Slc22a7,* and *Abcb1b* (transporters). Other genes more expressed in males than in females were *Rgn* and *Cacng5,* encoding for enzymes involved in calcium transport. 

### 2.2. Differentially Expressed Genes after OTA Treatment

Genes modulated by OTA (0.50 mg/kg b.w.) after 7 or 21 days of administration were analyzed in females. It was observed that the number of DEG was clearly time-dependent: 494 at 7 days and 2409 at 21 days (Table 3); 86% (426/494) DEG at 7 days were also altered at 21 days. Both time points had a similar number of up- and down-regulated genes, although down-regulation slightly tended to be the main effect (57% 7 days and 56% 21 days) (Table 3). Moreover, all genes showed the same direction of modulation after 7 and 21 days of OTA treatment. Thus, OTA-induced gene expression response was progressive and consistent over time in females.

Regarding the magnitude of OTA-induced gene expression changes, only a few genes showed a logFC value higher than 1.5 or lower than −1.5 and have been studied more in-depth: 32 at 7 days and 136 at 21 days (Table 3); 27 were common to both time points. Of all of them (141), 33 genes were significantly modified in females but not in males (Table S1 supplementary data). Regarding the list of genes with a high logFC value and a known function, it can be seen that they encoded enzymes (*Akr1b7*, *Adh6*, *Akr1c2*) or transporters (*Slc17a9*), and *Akr1b7* was consistently down-regulated at both time points (Table 4). 

Gene expression response in males was also time-dependent: 828 at 7 days and 2234 at 21 days (Table 3), but contrary to females, only 33% (277/828) of the genes altered at 7 days were also affected at 21 days. Moreover, at 7 days, up-regulation was the main effect (83%; 689/828), but at 21 days, almost the same number of genes were up- and down-regulated (up: 1106/down: 1128) (Table 3). In addition, some of these genes showed a different modification pattern at each time point: up-regulation after 7 days and down-regulation after 21 days. 

As in females, despite the high number of DEG, only 19 at 7 days or 136 at 21 days showed logFC higher than 1.5 or less than −1.5 (Table 3), and only nine genes were common to both time points. Of all of them (146), 27 genes were exclusively modified in males (Table S2 supplementary data). Regarding the list of genes with known functions, some of them encoded enzymes (*Cyp2c11*, *Dhrs7*, *Cyp2d1*, *Cyp2d5*, *Cyp24a1*) or transporters (*Slco1a1*, *Slc51b*, *Slc22a22*), and *Cyp2c11* was consistently down-regulated over time (Table 4). Other transporters genes (*Slc22a13*, *Slc5a10*) were significantly down-regulated in both sexes at 21 days (logFC < −2 in females and < −3 in males). 

## 3. Discussion

The aim of the present study was to determine the time-course of gene expression changes after subchronic oral OTA (0.50 mg/kg b.w.) administration to F344 rats and see if the response was different between sexes. Basal sex differences in kidney gene expression during the life cycle of F344 rats have been previously studied, and the most prominent differences have been observed in the range of 8 to 21 weeks of age [26]. The age of the animals of this study sacrificed after 7 and 21 days was 13- and 15-week old, respectively. They correspond to middle-aged adults, which is commonly used in toxicological studies. Our results basically agree with Kwekel et al. (2013) [26], who considered *Adh6, Akr1b7*, *Abcb1b* (overexpressed in females) and *Slco1a1*, *Dhrs7*, *Slc22a22*, *Cyp2c11,* and *Mlc1* (overexpressed in males) genes as the main contributors of sex differences. In our study, other genes coding enzymes or transporters were also found to be female-biased (*Akr1c2*, *Adh1*, *Slc17a9*, *Slc22a7*) or male-biased (*Slc51b*, *Sult1b1*, *Cyp2d1*, *Akr1c12*, *Cyp2d5*, *Cyp4a2*) (Table 2). Regarding genes qualified by FDA as preclinical biomarkers of kidney injury (*Gstp1*, *Fabp3,* and *Ntn1*), no sex differences were observed in our study. However, some discrepancies were found with Kwekel et al. (2013) [26]: *Tff3* and *Spp1* were both male-biased, while *Gstm1* did not show sex differences in our dataset. Overall, the results indicate that vehicle administration did not modify basal gene expression in control animals.

In the present study, the OTA dose administered to the animals was selected based on previous carcinogenic studies in rats and on some short-term toxicity studies. The aim was to reach a toxic effect in a 21 days repeated dose toxicity study, at doses near the range of OTA carcinogenic doses. The dose of 0.5 mg OTA/kg b.w. is within the range of 1.25×–2.4× doses inducing tumors after long-term daily dietary OTA exposure in 2 years carcinogenic studies: 0.4 mg/kg b.w. [6]; 0.3 mg/kg b.w. [23]; 0.21 mg/kg b.w. [5]. Some authors observed kidney histopathological alterations after 2 weeks of OTA oral dose (0.25, 0.5, 1, and 2 mg/kg b.w.); the effect was dose-dependent [25]. In the present study, gene expression changes were analyzed at the dose of 0.5 mg/kg b.w. because it was known that it produced some nephrotoxic effect after daily gavage to male Fisher rats in a 21 days toxicity study [24]. 

Regarding the time course of response to OTA treatment, a greater number of genes were differentially expressed after 21 days of OTA treatment than after 7 days, similar to what was found in males, administered with the same dose of OTA (0.50 mg/kg b.w.): 780 versus 1961 [24]. Besides, after 7 days and 12 months of OTA treatment of male F344 rats, a similar time-response was observed, but the number of DEGs at each time was not provided [23]. This finding could not be explained by the authors who recognized it was difficult to understand. In the present study, the comparison of gene response over time between males and females gave some differences: in females, the response was progressive and consistent with a slight tendency to gene expression down-regulation, whereas, in males, the total number of DEG was much higher than in females after 7 days; moreover, up-regulation was the predominant effect at 7 days, but at 21 days, an equal number of genes were up- and down-regulated. This data indicate that males had a different early response to OTA. 

The genes that were exclusively modified in treated females were all overexpressed in females of control groups aged 13 or 15 weeks (female-biased genes), and OTA treatment produced a decrease in their expression (Table 2 and Table 4). In males, all the genes that were modified by OTA, except *Ucp1*, *Thrsp,* and *Cyp24a1*, were also overexpressed in control males with respect to females (male-biased genes), and OTA treatment produced a decrease in their expression (Table 2 and Table 4). Thus, OTA treatment would apparently diminish the basal sex differences found in those genes. None of the genes were identified as DEG in the previous studies performed on male F344 rats, although some other phase I enzymes genes (*Akr7a3*, *Cyp2c*, *Cyp2d9*, *Cyp2d6*) or transporters genes (*Slc22a6*, *Slc21a1*, *Slc21a4*, *Slc2a5*, *Slc22a1*, *Slc22a2*) were also modulated in OTA-treated male F344 rats [23,24]. These results indicate that OTA treatment changed the gene expression of enzymes and transporters genes, some of them having a different basal level in males and females, and down-regulation was the main effect in both sexes.

Aldo-keto reductases (*Akr1b7*, *Akr1c2*) and alcohol dehydrogenases (*Adh6*) appeared as three of the most inhibited genes after OTA exposure, only in females. No information related to aldo-keto reductase activity with OTA has been found. However, these enzymes have been described to participate in the bioactivation/detoxification of several carcinogens [27,28]. Moreover, some alcohol dehydrogenases oxidize some of the less toxic metabolites of OTA [29]. In males, some genes of the great cytochrome P450 (CYP) family are modified after OTA treatment, and some of them have been related to its metabolism. In human hepatocytes, OTA up-regulated *CYP3A4*, *CYP2B6*, *CYP3A5*, *CYP2C9*, *CYP1A1*, *CYP1A2,* and it was suggested that OTA could activate PXR and Ahr, while CAR activation was not affected [30]. More recently, it was found that OTA suppressed PXR-mediated *CYP3A4* induction in primary cultures of human hepatocytes [31]. Much of the sex-biased metabolic response might be directly related to an upstream regulation via nuclear receptor signaling, but we could not confirm the hypothesis in this study. In our data set, *PXR/RXR* activation toxicity list appeared as significantly modulated in both sexes, and several enzyme genes were present in this list, such as *Cyp2c11*, *Cyp3a9*, *Aldh3a2*, *Aldh1a1* [32]. Marin-Kuan et al. (2006) found that many of the metabolism-related enzymes and transporters DEG, which were down-regulated in OTA-treated male rats, were under the transcriptional control of the hepatocyte nuclear factor 4 alpha (HNF4α) [23].

In microsomes expressing the human homolog of *Cyp2c11* (CYP2C9) incubated with the mycotoxin, OTA-DNA adducts were found [33]. According to this, it could be hypothesized that F344 males that overexpress Cyp2c11 at a basal level might produce high levels of reactive metabolites when first exposed to OTA and that this could be a key event necessary to initiate a carcinogenic response, even if OTA treatment tends to decrease this isoform over time. The specific role of other CYPs, such as *Cyp2d1*, *Cyp2d5,* or *Cyp24a1,* should also be taken into account; all of them are DEG only in males. Cyp2d1 is considered as the rat ortholog of human CYP2D6, an enzyme presenting many human polymorphisms [34]. In Dark-Agouti (DA) male and female rats, considered to be extensive and poor debrisoquine (CYP2D6 substrate) metabolizers respectively, male DA rats were the most susceptible to OTA-induced tumorigenesis, while DA females were resistant (Table 1); thus, OTA tumor susceptibility was related to CYP2D6 metabolism [35]. A high *Cyp2d1* expression in male rats, which was found in this study, might also contribute to additional OTA-DNA adducts after initial OTA exposures. On the contrary, in OTA-treated NIH/3T3 cells stably expressing the human ortholog CYP2D6, no increase of mutation frequency was observed [36]. On the other hand, the *Cyp24a1* human ortholog (CYP24A1) is known to play a role in calcium homeostasis and the vitamin D endocrine system by degrading 1,25-dihydroxyvitamin D3 to the physiologically active form of vitamin D3. There is increasing evidence that vitamin D metabolites influence carcinogenesis, and, indeed, CYP24A1 has been found to be up-regulated in tissues of patients with renal cell carcinoma [37] and has been proposed as a candidate oncogene for colorectal tumorigenesis [38]. 

Several transporters genes have been found to be down-regulated after OTA treatment: *Slc17a9* in females; *Slco1a1* (Oat) and *Slc51b* and *Slc22a22* (Oatp) in males (see Table 4). These results correlate with other previously obtained in the same kidney samples by RT-qPCR: after 21 days of 0.50 mg/kg OTA treatment, Oatp1 and several OAT transporters (Oat1, Oat2, Oat3, Oat5, and Oat8) were strongly down-regulated in males [39]. 

One mechanistic hypothesis for OTA toxicity is the production of free radicals and the resultant activation of redox-regulated transcription factors and antioxidant enzymes catalase (CAT), superoxide dismutase (SOD), or glutathione peroxidase (GPx) [11]. Almost all gene expression studies carried out with OTA reported oxidative stress deregulation either in vitro or in vivo [22]. Moreover, the impairment of cellular defense mechanisms by down-regulation of many Nrf2-regulated genes that would conduct to an oxidative stress condition has been proposed as one of the mechanisms that could contribute to OTA carcinogenicity [40]. More concretely, OTA has been proposed to inhibit Nrf2, a molecule that drives the transcription of genes involved in glutathione synthesis and recycling, phase II metabolism, and the reduction of oxygen species and quinones [41]. In our dataset, Nrf2 was not differentially expressed after OTA treatment, but many genes related to antioxidant cell defenses were down-regulated; some of them under the control of Nrf2 [32]. However, at the phenotypic level, in kidney samples obtained from the same animals used in the present study, no significant differences between sexes or with respect to untreated animals were observed in oxidative stress indicators (total glutathione (tGSH), glutathione disulfide (GSSG), glutathione-S-transferase (GST), and SOD) or in the oxidative damage to DNA at any time point [42]. Besides, in kidney tissue of male Wistar rats treated with 1 or 4 mg/kg of OTA for 7 days, oxidative DNA damage or significant changes in oxidative stress biomarkers (reactive oxygen species (ROS), SOD, GSH, and malondialdehyde (MDA)) were not found [43]. In more recent studies, biochemical and histopathological alterations in kidney were observed in male Sprague-Dawley rats fed with OTA 0.5 mg/kg b.w. for 14 days; assuming an oxidative stress mechanism, a protective effect of different antioxidants (Delta-tocotrienol, curcumin, or red orange and lemon extracts) was referred by the authors [44,45,46]. The rat strain or OTA vehicles used (dimethyl sulfoxide (DMSO), olive oil, bicarbonate), the shorter time treatment, a possible heterogeneity in the phenotypical information obtained in systemic general toxicity studies, and the validation of the biochemical oxidative biomarkers used are all factors that may account for these differences. In accordance with this, stronger signs of toxicity were also observed in kidney histological preparations of F344 rats after 7 days of OTA treatment than after 21 days of OTA administration [39]. 

In summary, it can be concluded that after subchronic OTA treatment, (i) there was a time course of gene expression in kidney tissue, with more DEG at 21 days than at 7 days, (ii) the transcriptomic time course was different in males and females with respect to the number of DEG and the direction of expression (up or down), (iii) greater sex differences were observed at short treatment time (7 days), (iv) the main sex differences were found with metabolizing enzymes and transporters, (v) some of the DEG showing sex differences was also differentially expressed in both sexes at the basal level.

## 4. Materials and Methods

### 4.1. Chemicals

Ochratoxin A was obtained in powder from Sigma-Aldrich (Steinheim, Germany), dissolved in NaHCO_3_ (0.1 M pH 7.4) (Sigma-Aldrich; Steinheim, Germany), and stored at −20 °C until use.

### 4.2. Experimental Design

The kidney samples were obtained in a previous study that was approved by the Ethics Committee on Animal Experimentation of the University of Navarra [36]. Briefly, eleven-week-old male and female Fisher 344 (F344/IcoCrl) rats weighing 260.93 ± 12.0 and 157.98 ± 8.52 g, respectively, on the day of arrival, were received at the Charles Rivers Laboratories (France). Females were not synchronized for the estrous cycle. All animals were randomly distributed into groups of 6 animals (males or females). They were housed in polycarbonate cages with stainless steel covers, under standard conditions (temperature 22 ± 3 °C, humidity 50 ± 20%, 12 h light/dark cycle), and provided with sterile food (Harlan) and water *ad libitum*.

After a week of acclimatization, animals were daily gavaged (maximum volume of 1 mL/0.1 kg b.w.) with 0.50 mg of OTA/kg b.w. or the vehicle (NaHCO_3_ 0.1 M pH 7.4) (control group) for 7 or 21 days (4 male groups and 4 female groups). Rats were daily weighted for adjusted administration, while food and water consumption were measured once a week. After 24 h of the last administration, animals were euthanized by decapitation. 

For this study, the left kidney of each animal was removed and longitudinally cut into two halves. Each half was then divided into four pieces and flash-frozen in liquid nitrogen for nucleic acid extractions. All the dissection material was cleaned with water and rinsed with ethanol after each animal necropsy in order to prevent contamination between samples. All frozen tissues were stored at −80 °C until use. RNA isolation was carried out using two different pieces of left kidneys that contained all structures (cortex, medulla, and papilla).

### 4.3. RNA Isolation

For gene expression analysis, 3–4 samples per treatment and sex were used. Total RNA was isolated from approximately 50 mg of frozen kidneys according to the TRIzol^®^ manufacturer’s protocol (Invitrogen™, Carlsbad, CA, USA). For that purpose, kidney samples were homogenized in TRIzol^®^ (50 mg/mL) with a T25 Ultra-turrax Digital High Speed Homogenizer (IKA^®^, Staufen, Germany). Extracted RNA (100 µg) was purified according to AllPrep DNA/RNA kit’s protocol (Qiagen, Hilden, Germany) and dissolved in RNase-free water. The purity and quality of extracted RNA were first evaluated spectrophotometrically (SmartSpec™ Plus, Bio-Rad) by measuring the optical density at 260 nm (OD_260_ = 44 µg of RNA) and then, after purification, with Experion Bioanalyzer (Bio-Rad Laboratories, Hercules, CA, USA) using Experion™ STDSens RNA Chips (Bio-Rad). All the samples showed an A260/A280 ratio between 1.8 and 2.0 and an average RNA quality indicator (RQI) value of 8.7 ± 0.7 (standard deviation, SD).

### 4.4. Gene Expression Experiments (Microarrays)

Gene expression analyses were performed by the Proteomics, Genomics, and Bioinformatics Unit of the Center for Applied Medical Research (CIMA) from the University of Navarra.

The sense cDNA was prepared from 300 ng of total RNA using the Ambion^®^ (Austin, TX, USA) WT Expression Kit. The sense strand cDNA was then fragmented and biotinylated with the Affymetrix GeneChip^®^ (Santa Clara, CA, USA) WT Terminal Labeling Kit (PN 900671). Labeled sense cDNA was hybridized to GeneChip^®^ Rat Gene 2.0 ST Array (Affymetrix) according to the manufacturer protocols and using GeneChip^®^ Hybridization, Wash, and Stain Kit. Gene chips were scanned with the Affymetrix GeneChip^®^ Scanner 3000.

### 4.5. Gene Expression Data Analysis

Both background correction and normalization were done using RMA (Robust Multichip Average) algorithm [47]. After a quality study of samples and outlier detection, all samples were considered for further analysis. Then, a filtering process was performed to eliminate low expression probe sets. Applying the criterion of an expression value (log_2_) greater than 5 in 2 samples in at least one of the experimental conditions, 24,947 and 27,790 probe sets were selected for statistical analysis of 7- and 21-day OTA toxicity studies, respectively. R/Bioconductor [48] was used for pre-processing and statistical analysis.

LIMMA (Linear Models for Microarray Data) [49] was used to find out the probe sets that showed significant differential expression between experimental conditions. For that purpose, two different approaches were used. Firstly, in order to determine basal sex differences, at each time point (7 or 21 days), control animals of both sexes were compared (males vs. females, MC vs. FC). Then, to determine the OTA treatment effect in each sex, treated vs. control groups were compared per time point and sex. Probe sets were considered significant using a B statistic cut-off of B > 0 for all contrasts and identified as differentially expressed genes (DEG) in each comparison. The total number of DEGs of each contrast was calculated, taking into account the number of Affymetrix’s annotated genes.

## Figures and Tables

**Table 1:** Ochratoxin A carcinogenicity studies in male and female rodents: incidence of benign (adenomas) and malign (carcinomas) cell tumors.

Reference	Species and Strain Dose	Sex	Number of Animals with Tumors/Number of Animals Examined at Each Dose	
Bendele et al., 1985 [4]	B6C3F mice 0, 1, 40 ppm		Renal adenoma	Renal carcinoma
		M	0/50, 0/47, 26/50	0/50, 0/47, 14/50
		F	0/47, 0/45, 0/49	0/47, 0/45, 0/49
NTP, 1989 [5]	F344 rats 0, 21, 70, 210 µg/kg		Renal adenoma	Renal carcinoma
		M	1/50, 1/51, 6/51, 10/50	0/50, 0/51, 16/51, 30/50
		F	0/50, 0/51, 1/50, 5/50	0/50, 0/51, 1/30, 3/50
Castegnaro et al., 1998 [6]	DA and Lewis rats 0, 400 µg/kg		Renal adenoma	Renal carcinoma
		M DA	0/10, 5/20	0/10, 20/20
		F DA	0/10, 0/40	0/10, 0/40
		M Lewis	0/10, 0/20	0/10, 10/20
		F Lewis	0/10, 0/20	0/10, 5/20

DA, Dark-Agouti; F, female; M, male.

**Table 2:** Gene expression in male and female F344 rats administered with the vehicle (NaHCO_3_) for 7 or 21 days (controls). Gene expression in males was compared with gene expression in females, and DEG (differentially expressed genes) with logFC (logarithm Fold Change) higher or lower than |1.50|, common to both time points (13- and 15-week-old F344 rats) is presented. Genes overexpressed in males (positive logFC values) were considered male-biased genes, and genes overexpressed in females (negative logFC values) were considered female-biased genes.

Gene Name	Gene Description	LogFC	
		13 Weeks	15 Weeks
Male-biased genes			
Dhrs7	dehydrogenase/reductase (SDR family) member 7	6.85	6.97
LOC100364391	dehydrogenase/reductase (SDR family) member 7-like	6.43	6.24
Slco1a1	solute carrier organic anion transporter family, member 1a1	6.34	6.99
Slc22a22	solute carrier family 22 (organic cation transporter), member 22	5.91	5.80
Cyp2c11	cytochrome P450, subfamily 2, polypeptide 11	5.18	4.92
Eif2s3y	eukaryotic translation initiation factor 2, subunit 3, structural gene Y-linked	4.12	4.25
Slc51b	solute carrier family 51, beta subunit	4.06	3.61
Gc	group specific component	3.92	3.31
Rgn	regucalcin (senescence marker protein-30)	3.47	3.06
Ddx3	DEAD (Asp-Glu-Ala-Asp) box polypeptide 3	3.45	3.65
Mybl1	myeloblastosis oncogene-like 1	3.45	3.29
LOC102550584	hornerin-like	2.85	2.89
Sult1b1	sulfotransferase family, cytosolic, 1B, member 1	2.71	3.05
Tff3	trefoil factor 3, intestinal	2.48	1.65
Cyp2d1	cytochrome P450, family 2, subfamily d, polypeptide 1	2.36	2.21
Cacng5	calcium channel, voltage-dependent, gamma subunit 5	2.36	2.14
Akr1c12l1	aldo-keto reductase family 1, member C12-like 1	2.35	2.11
Rarres1	retinoic acid receptor responder (tazarotene induced) 1	2.33	1.70
Anxa13	annexin A13	2.23	2.41
Melk	maternal embryonic leucine zipper kinase	2.01	1.74
Cyp2d5	cytochrome P450, family 2, subfamily d, polypeptide 5	2.01	2.23
Cndp1	carnosine dipeptidase 1 (metallopeptidase M20 family)	1.95	1.80
Prlr	prolactin receptor	1.93	2.03
LOC500124	similar to RIKEN cDNA 4921507P07	1.92	1.70
Gucy1b2	guanylate cyclase 1, soluble, beta 2	1.90	1.90
Cyp4a2	cytochrome P450, family 4, subfamily a, polypeptide 2	1.85	1.98
Pecr	peroxisomal trans-2-enoyl-CoA reductase	1.83	2.00
Hpgd	hydroxyprostaglandin dehydrogenase 15 (NAD)	1.81	1.73
Mapk10	mitogen activated protein kinase 10	1.80	1.84
Akr1c12	aldo-keto reductase family 1, member C12	1.72	2.11
Nkd2	naked cuticle homolog 2 (Drosophila)	1.67	1.50
RGD1564999	similar to isopentenyl-diphosphate delta isomerase 2	2.22	2.78
Pzp	pregnancy-zone protein	1.62	2.08
Tmem236	transmembrane protein 236	1.54	1.67
Oosp1	oocyte secreted protein 1	1.54	1.80
Mlc1	megalencephalic leukoencephalopathy with subcortical cysts 1	1.50	1.51
Female-biased genes			
Adh6	alcohol dehydrogenase 6 (class V)	−6.17	−5.83
Akr1b7	aldo-keto reductase family 1, member B7	−5.25	−5.72
Ly6al	lymphocyte antigen 6 complex, locus A-like	−3.69	−4.21
Akr1c2	aldo-keto reductase family 1, member C2	−2.80	−2.96
Slc17a9	solute carrier family 17 (vesicular nucleotide transporter), member 9	−2.50	−2.37
Adh1	alcohol dehydrogenase 1 (class I)	−2.33	−1.79
Cmtm2a	CKLF-like MARVEL transmembrane domain containing 2A	−1.83	−2.17
Col17a1	collagen, type XVII, alpha 1	−1.82	−2.13
Spetex-2H	Spetex-2H protein	−1.77	−2.55
Slc22a7	solute carrier family 22 (organic anion transporter), member 7	−1.73	−2.44
Col24a1	collagen, type XXIV, alpha 1	−1.70	−2.45
Abcb1b	ATP-binding cassette, subfamily B (MDR/TAP), member 1B	−1.68	−1.85
Akap17b	A kinase (PRKA) anchor protein 17B	−1.57	−2.15
Tspan8	tetraspanin 8	−1.52	−1.89
Srsf12	serine/arginine-rich splicing factor 12	−1.50	−1.67

**Table 3:** Number of DEG (B-value > 0) after OTA treatment (0.50 mg/kg b.w.) for 7 or 21 days in males and females.

Treatment	DEG	Males	Females
7 days	Up-regulated (log FC > 0)	689	213
	Down-regulated (log FC < 0)	139	281
	Total	828	494
	Log FC > or <1.5	19	32
21 days	Up-regulated (log FC > 0)	1106	1067
	Down-regulated (log FC < 0)	1128	1342
	Total	2234	2409
	Log FC > or <1.5	136	136

**Table 4:** DEG with known function found in kidney tissue of F344 rats after OTA treatment (7 or 21 days), with logFC higher or lower than |1.5|, that were exclusively modified in females or in males. (See complete lists in Tables S1 and S2 of supplementary data).

	Gene Name	Gene Description	LogFC 7 Days	LogFC 21 Days
Females	Akr1b7	aldo-keto reductase family 1, member B7	−2.99	−5.37
	Adh6	alcohol dehydrogenase 6 (class V)		−2.30
	Akr1c2	aldo-keto reductase family 1, member C2		−2.10
	Slc17a9	solute carrier family 17, member 9		−2.12
Males	Ucp1	uncoupling protein 1 (mitochondrial, proton carrier)	−2.72	
	Thrsp	thyroid hormone responsive	−2.55	
	Cyp2c11	cytochrome P450, subfamily 2, polypeptide 11	−1.96	−4.43
	Slco1a1	solute carrier organic anion transporter family, member 1a1		−5.26
	Dhrs7	dehydrogenase/reductase (SDR family) member 7		−4.49
	Cyp2d1	cytochrome P450, family 2, subfamily d, polypeptide 1		−2.35
	LOC100364391	dehydrogenase/reductase (SDR family) member 7-like		−2.27
	Cyp2d5	cytochrome P450, family 2, subfamily d, polypeptide 5		−2.13
	Slc51b	solute carrier family 51, beta subunit		−2.02
	Slc22a22	solute carrier family 22 (organic cation transporter), member 22		−1.53
	Cyp24a1	cytochrome P450, family 24, subfamily a, polypeptide 1		1.54

## Data Availability

Not applicable.

## Supplementary Materials

The following are available online at https://www.mdpi.com/2072-6651/13/3/177/s1, Table S1: DEG obtained in F344 rats after OTA treatment (7 or 21 days) with logFC higher or lower than |1.5| that were exclusively modified in females, Table S2: DEG obtained in F344 rats after OTA treatment (7 and 21 days) with logFC higher or lower than |1.5| that were exclusively modified in males.

## References

[1] European Food Safety Authority (EFSA) (2006). Contaminants in the food chain on a request from the Commission related to ochratoxin A (OTA) in food. EFSA J..

[2] Damiano S., Longobardi C., Andretta E., Prisco F., Piegari G., Squillacioti C., Montagnaro S., Pagnini F., Badino P., Florio S. (2021). Antioxidative Effects of Curcumin on the Hepatotoxicity Induced by Ochratoxin A in Rats. Antioxidants.

[3] Lock E.A., Hard G.C. (2004). Chemically induced renal tubule tumors in the laboratory rat and mouse: Review of the NCI/NTP database and categorization of renal carcinogens based on mechanistic information. Crit. Rev. Toxicol..

[4] Bendele A.M., Carlton W.W., Krogh P., Lillehoi E.B. (1985). Ochratoxin A carcinogenesis in the (C57BL/6J X C3H) F1 mouse. J. Natl. Cancer Inst..

[5] National Toxicology Program (NTP) (1989). Toxicology and carcinogenesis studies of ochratoxin A (CAS No. 303-47-9) in F344/N Rats (Gavage studies). Natl. Toxicol. Program Tech. Rep. Ser..

[6] Castegnaro M., Mohr U., Pfohl-Leszkowicz A., Esteve J., Steinmann J., Tillmann T., Michelon J., Bartsch H. (1998). Sex- and strain-specific induction of renal tumors by ochratoxin A in rats correlates with DNA adduction. Int. J. Cancer.

[7] Stoev S.D. (2021). Follow up long term preliminary studies on carcinogenic and toxic effects of ochratoxin A in rats and the putative protection of phenylalanine. Toxicon.

[8] International Agency for Research in Cancer (IARC) (1993). IARC Monographs on the evaluation of carcinogenic risks to humans. Some naturally occurring substances: Food items and constituents, heterocyclic aromatic amines and mycotoxins. IARC Monogr..

[9] National Toxicology Program (NTP) (2016). Report on Carcinogens.

[10] Malir F., Ostry V., Pfohl-Leszkowicz A., Malir J., Toman J. (2016). Ochratoxin A: 50 Years of Research. Toxins.

[11] Tao Y., Xie S., Xu F., Liu A., Wang Y., Chen D., Pan Y., Huang L., Peng D., Wang X. (2018). Ochratoxin A: Toxicity, oxidative stress and metabolism. Food Chem. Toxicol..

[12] Zhu L., Zhang B., Dai Y., Li H., Xu W. (2017). A Review: Epigenetic Mechanism in Ochratoxin A Toxicity Studies. Toxins.

[13] World Health Organisation (WHO) (2008). Safety evaluation of certain food additives and contaminants. WHO Food Addit. Ser..

[14] Pfohl-Leszkowicz A., Manderville R.A. (2007). Ochratoxin A: An overview on toxicity and carcinogenicity in animals and humans. Mol. Nutr. Food Res..

[15] Pfohl-Leszkowicz A., Manderville R.A. (2012). An update on direct genotoxicity as a molecular mechanism of ochratoxin a carcinogenicity. Chem. Res. Toxicol..

[16] Marin-Kuan M., Cavin C., Delatour T., Schilter B. (2008). Ochratoxin A carcinogenicity involves a complex network of epigenetic mechanisms. Toxicon.

[17] Marin-Kuan M., Ehrlich V., Delatour T., Cavin C., Schilter B. (2011). Evidence for a role of oxidative stress in the carcinogenicity of ochratoxin A. J. Toxicol..

[18] Rumora L., Zanic-Grubisic T. (2009). A journey through mitogen-activated protein kinase and ochratoxin A interactions. Arh. Hig. Rada. Toksikol..

[19] Mally A. (2012). Ochratoxin A and mitotic disruption: Mode of action analysis of renal tumor formation by ochratoxin A. Toxicol. Sci..

[20] Kuiper-Goodman T., Hilts C., Billiard S.M., Kiparissis Y., Richard I.D., Hayward S. (2010). Health risk assessment of ochratoxin A for all age-sex strata in a market economy. Food Addit. Contam. Part A Chem. Anal. Control Expo Risk Assess..

[21] Afshari C.A., Hamadeh H.K., Bushel P.R. (2011). The evolution of bioinformatics in toxicology: Advancing toxicogenomics. Toxicol. Sci..

[22] Vettorazzi A., van Delft J., López de Cerain A. (2013). A review on ochratoxin A transcriptomic studies. Food Chem. Toxicol..

[23] Marin-Kuan M., Nestler S., Verguet C., Bezencon C., Piguet D., Mansourian R., Holzwarth J., Grigorov M., Delatour T., Mantle P. (2006). A toxicogenomics approach to identify new plausible epigenetic mechanisms of ochratoxin A carcinogenicity in rat. Toxicol. Sci..

[24] Arbillaga L., Vettorazzi A., Gil A.G., van Delft J.H., García-Jalón J.A., López de Cerain A. (2008). Gene expression changes induced by ochratoxin A in renal and hepatic tissues of male F344 rat after oral repeated administration. Toxicol. Appl. Pharmacol..

[25] Mally A., Völkel W., Amberg A., Kurz M., Wanek P., Eder E., Hard G., Dekant W. (2005). Functional, biochemical, and pathological effects of repeated oral administration of ochratoxin A to rats. Chem. Res. Toxicol..

[26] Kwekel J.C., Desai V.G., Moland C.L., Vijay V., Fuscoe J.C. (2013). Sex differences in kidney gene expression during the life cycle of F344 rats. Biol. Sex Differ..

[27] Jin Y., Penning T.M. (2007). Aldo-keto reductases and bioactivation/detoxication. Annu. Rev. Pharmacol. Toxicol..

[28] Knight L.P., Primiano T., Groopman J.D., Kensler T.W., Sutter T.R. (1999). cDNA cloning, expression and activity of a second human aflatoxin B1-metabolizing member of the aldo-keto reductase superfamily, AKR7A3. Carcinogenesis.

[29] Syvertsen C., Størmer F.C. (1983). Oxidation of two hydroxylated ochratoxin A metabolites by alcohol dehydrogenase. Appl. Environ. Microbiol..

[30] Ayed-Boussema I., Pascussi J.M., Zaied C., Maurel P., Bacha H., Hassen W. (2012). Ochratoxin A induces CYP3A4, 2B6, 3A5, 2C9, 1A1, and CYP1A2 gene expression in primary cultured human hepatocytes: A possible activation of nuclear receptors. Drug Chem. Toxicol..

[31] Doricakova A., Vrzal R. (2015). A food contaminant ochratoxin A suppresses pregnane X receptor (PXR)-mediated CYP3A4 induction in primary cultures of human hepatocytes. Toxicology.

[32] Vettorazzi A., Pastor L., Guruceaga E., López de Cerain A. (2019). Sex-dependent gene expression after ochratoxin A insult in F344 rat kidney. Food Chem. Toxicol..

[33] El Adlouni C., Pinelli E., Azémar B., Zaoui D., Beaune P., Pfohl-Leszkowicz A. (2000). Phenobarbital increases DNA adduct and metabolites formed by ochratoxin A; role of CYP2C9 and microsomal glutathione-S-transferase. Environ. Mol. Mutagen..

[34] Martignoni M., Groothuis G.M., de Kanter R. (2006). Species differences between mouse, rat, dog, monkey and human CYP-mediated drug metabolism, inhibition and induction. Expert Opin. Drug Metab. Toxicol..

[35] Pfohl-Leszkowicz A., Pinelli E., Bartsch H., Mohr U., Castegnaro M. (1998). Sex- and strain-specific expression of cytochrome P450s in ochratoxin A-induced genotoxicity and carcinogenicity in rats. Mol. Carcinog..

[36] De Groene E.M., Hassing I.G., Blom M.J., Seinen W., Fink-Gremmels J., Horbach G.J. (1996). Development of human cytochrome P450-expressiong cell lines: Application in mutagenicity testing of ochratoxin A. Cancer Res..

[37] Urbschat A., Paulus P., von Quernheim Q.F., Brück P., Badenhoop K., Zeuzem S., Ramos-Lopez E. (2013). Vitamin D hydroxylases CYP2R1, CYP27B1 and CYP24A1 in renal cell carcinoma. Eur. J. Clin. Investig..

[38] Horváth H.C., Lakatos P., Kósa J.P., Bácsi K., Borka K., Bises G., Nittke T., Hershberger P.A., Speer G., Kállay E. (2010). The candidate oncogene CYP24A1: A potential biomarker for colorectal tumorigenesis. J. Histochem. Cytochem..

[39] Pastor L., Vettorazzi A., Enciso J.M., González-Peñas E., García-Jalón J.A., Monreal J.I., López de Cerain A. (2018). Sex differences in ochratoxin a toxicity in F344 rats after 7 and 21 days of daily oral administration. Food Chem. Toxicol..

[40] Limonciel A., Jennings P. (2014). A review of the evidence that ochratoxin A is an Nrf2 inhibitor: Implications for nephrotoxicity and renal carcinogenicity. Toxins.

[41] Cavin C., Delatour T., Marin-Kuan M., Holzhäuser D., Higgins L., Bezencon C., Guignard G., Junod S., Richoz-Pavot J., Gremaud E. (2007). Reduction in antioxidant defenses may contribute to ochratoxin A toxicity and carcinogenicity. Toxicol. Sci..

[42] Enciso J.M., López de Cerain A., Pastor L., Azqueta A., Vettorazzi A. (2018). Is oxidative stress involved in the sex-dependent response to ochratoxin A renal toxicity?. Food Chem. Toxicol..

[43] Zhu L., Yu T., Qi X., Gao J., Huang K., He X., Luo H., Xu W. (2016). Limited Link between Oxidative Stress and Ochratoxin A-Induced Renal Injury in an Acute Toxicity Rat Model. Toxins.

[44] Damiano S., Navas L., Lombari P., Montagnaro S., Forte I.M., Giordano A., Florio S., Ciarcia R. (2018). Effects of δ-tocotrienol on ochratoxin A-induced nephrotoxicity in rats. J. Cell. Physiol..

[45] Damiano S., Andretta E., Longobardi C., Prisco F., Paciello O., Squillacioti C., Mirabella N., Florio S., Ciarcia R. (2020). Effects of Curcumin on the Renal Toxicity Induced by Ochratoxin A in Rats. Antioxidants.

[46] Damiano S., Iovane V., Squillacioti C., Mirabella N., Prisco F., Ariano A., Amenta M., Giordano A., Florio S., Ciarcia R. (2020). Red orange and lemon extract prevents the renal toxicity induced by ochratoxin A in rats. J. Cell. Physiol..

[47] Irizarry R.A., Bolstad B.M., Collin F., Cope L.M., Hobbs B., Speed T.P. (2003). Summaries of affymetrix genechip probe level data. Nucleic Acids Res..

[48] Gentleman V., Carey S., Dudoit R., Irizarry R.A., Huber W. (2005). Bioinformatics and Computational Biology Solutions Using R and Bioconductor.

[49] Smyth G.K. (2004). Linear models and empirical Bayes methods for assessing differential expression in microarray experiments. Stat. Appl. Genet. Mol. Biol..

