# Supplementary material for: Time Course of Renal Transcriptomics after Subchronic Exposure to Ochratoxin A in Fisher Rats
Source: Toxins (Basel). 2021 Feb 26;13(3):177. doi: 10.3390/toxins13030177 (PMC7996782; doi:10.3390/toxins13030177)
Supplement: Supplementary file 1 [file toxins-13-00177-s001.pdf]

Time Course of Renal Transcriptomics after Subchronic Exposure to Ochratoxin A in Fisher Rats

Laura Pastor, Ariane Vettorazzi, Elizabeth Guruceaga and López de Cerain A

Table S1. DEG obtained in F344 rats after OTA treatment (7 or 21 days) with logFC higher or lower than ǀ1.5ǀ that were exclusively modified in females.

Gene Name	Gene Description	LogFC	
		7 days	21 days
	**7 & 21 days**		
Akr1b7	aldo-keto reductase family 1, member B7	−2.99	−5.37
Cmtm2a	CKLF-like MARVEL transmembrane domain containing 2A	−1.61	−1.96
	**7 days**		
Cxcl13	chemokine (C-X-C motif) ligand 13	1.52	
Bcl6	B-cell CLL/lymphoma 6	1.60	
Alox15b	arachidonate 15-lipoxygenase, type B	1.70	
	**21 days**		
Nxpe2	neurexophilin and PC-esterase domain family, member 2		−3.44
Spetex-2G	Spetex-2G protein		−3.11
Spetex-2H	Spetex-2H protein		−3.09
LOC102557499	uncharacterized LOC102557499		−3.02
Spetex-2D	Spetex-2D protein		−2.93
LOC102547212	disks large homolog 5-like		−2.76
Spetex-2E	Spetex-2E protein		−2.69
Spetex-2F	Spetex-2F protein		−2.54
LOC102547093	uncharacterized LOC102547093		−2.54
LOC102549465	disks large homolog 5-like		−2.52
Akap17b	A kinase (PRKA) anchor protein 17B		−2.50
Ly6i	lymphocyte antigen 6 complex, locus I		−2.45
LOC102546376	disks large homolog 5-like		−2.44
Adh6	alcohol dehydrogenase 6 (class V)		−2.30
Slc17a9	solute carrier family 17 (vesicular nucleotide transporter), member 9		−2.12
Akr1c2	aldo-keto reductase family 1, member C2		−2.10
Cntnap4	contactin associated protein-like 4		−2.05
LOC498470	similar to Spetex-2C protein		−2.01
Unc93a	unc-93 homolog A (C. elegans)		−1.89
LOC498465	similar to RIKEN cDNA 1700001F09		−1.84
LOC680656	hypothetical protein LOC680656		−1.76
Cnr1	cannabinoid receptor 1 (brain)		−1.76
Mall	mal, T-cell differentiation protein-like		−1.74
Col24a1	collagen, type XXIV, alpha 1		−1.68
Col17a1	collagen, type XVII, alpha 1		−1.57
Sectm1b	secreted and transmembrane 1B		1.76
Fkbp5	FK506 binding protein 5		2.03
Mybl1	myeloblastosis oncogene-like 1		2.34

Table S2. DEG obtained in F344 rats after OTA treatment (7 and 21 days) with logFC higher or lower than |1.5| that were exclusively modified in males.

Gene Name	Gene Description	LogFC	
		**7 days**	**21 days**
	**7 & 21 days**		
Cyp2c11	cytochrome P450, subfamily 2, polypeptide 11	−1.96	−4.43
	**7 days**		
Ucp1	uncoupling protein 1 (mitochondrial, proton carrier)	−2.72	
Thrsp	thyroid hormone responsive	−2.55	
Fabp4	fatty acid binding protein 4, adipocyte	−1.74	
Cml2	camello-like 2	2.23	
	**21 days**		
Slco1a1	solute carrier organic anion transporter family, member 1a1		−5.26
Dhrs7	dehydrogenase/reductase (SDR family) member 7		−4.49
LOC102550584	hornerin-like		−3.18
RGD1564999	similar to isopentenyl-diphosphate delta isomerase 2		−2.84
Cyp2d1	cytochrome P450, family 2, subfamily d, polypeptide 1		−2.35
LOC100364391	dehydrogenase/reductase (SDR family) member 7-like		−2.27
Cml3	camello-like 3		−2.15
Cyp2d5	cytochrome P450, family 2, subfamily d, polypeptide 5		−2.13
Gc	group specific component		−2.07
Slc51b	solute carrier family 51, beta subunit		−2.02
Oosp1	oocyte secreted protein 1		−1.92
Gucy1b2	guanylate cyclase 1, soluble, beta 2		−1.74
Tmem236	transmembrane protein 236		−1.71
Anxa13	annexin A13		−1.69
Nefm	neurofilament, medium polypeptide		−1.57
Mlc1	megalencephalic leukoencephalopathy with subcortical cysts 1		−1.57
Slc22a22	solute carrier family 22 (organic cation transporter), member 22		−1.53
Egr1	early growth response 1		1.53
Cyp24a1	cytochrome P450, family 24, subfamily a, polypeptide 1		1.54
S100g	S100 calcium binding protein G		1.58
Fam129a	family with sequence similarity 129, member A		1.66
Clmp	CXADR-like membrane protein		1.81
